# Impact of Minimal Steroid Doses on Post‐Transplant Growth in Pediatric Kidney Recipients, a Retrospective Observational Study

**DOI:** 10.1111/petr.70251

**Published:** 2025-12-21

**Authors:** Ochoa‐García Carolina Lucia, Villegas‐Arbeláez Esteban, Peláez‐Ortiz Juan Camilo, Serna‐Campuzano Angelica, Rojas‐Rosas Luisa Fernanda, Serna‐Higuita Lina Maria, Isaza‐López Maria Carolina

**Affiliations:** ^1^ Pediatric Department University of Antioquia Medellín Colombia; ^2^ Clinica Somer Rionegro Colombia; ^3^ Pediatric Department Universidad Remington Medellin Colombia; ^4^ Research Group in Clinical Epidemiology and Pediatrics Hospital Pablo Tobon Uribe Medellín Colombia; ^5^ Institut de Recerca Sant Pau Barcelona España; ^6^ Faculty of Medicine Universidad CES Medellín Colombia; ^7^ Pontificia Bolivariana University Medellín Colombia; ^8^ Hospital San Vicente de Paul Rionegro Colombia; ^9^ Hospital General de Medellin Colombia; ^10^ University Hospital Tübingen Tubingen Germany

**Keywords:** body height, glucocorticoids, growth disorders, kidney transplant, pediatrics

## Abstract

**Background:**

Despite the known impact of steroid therapy on growth after kidney transplantation (KTx), steroids remain widely used in pediatric KTx recipients. Evidence on the effect on linear growth at minimal doses of steroids (6 mg/m^2^ or 0.2 mg/kg/day) is limited. This study evaluated whether low doses of steroid therapy are associated with post‐transplant growth failure.

**Methods:**

Single‐center retrospective cohort study. In total, 44 pediatric KTx recipients from 2005 to 2024 were collected. Differences in *Z*‐Score and height before 6 months and after 6 and 12 months of KTx were assessed using Friedman rank tests. Linear mixed models were used to analyze the longitudinal effect of steroid therapy on growth outcomes.

**Results:**

The mean age at KTx was 11 years (SD ± 3.8). Median prednisolone doses at 3 and 6 months after KTx were 0.23 (p25–75: 0.18–0.41) and 0.21 mg/k/day (0.14–0.28), respectively. The mean‐height *Z*‐score improved from −2.35 (SD ± 1.30) to 1.82 (SD ± 1.23) one year post‐KTx. The percentage of patients with short stature (height *Z*‐score ≤ −2SD) decreased from 65.1% at baseline (KTx) to 54.8% and 52.3% after 6 and 12 months, respectively. In multivariable analysis, time follow‐up and age at KTx were associated with post‐transplant growth. Steroid therapy shows a trend to reduce body height, but the interaction was not significant.

**Conclusion:**

This single‐center study found that pediatric KTx recipients receiving low‐dose steroid therapy showed post‐transplant improvements in height and *Z*‐scores. Notably, the use of low‐dose steroids did not significantly impair growth, although a slight downward trend in *Z* scores was noted.

## Graphical abstract

In our study of 44 pediatric kidney transplant recipients, low‐dose corticosteroid therapy was associated with improvements in post‐transplant height and height *Z*‐scores over 1 year. Growth was not significantly impaired by low‐dose steroids, although a slight downward trend in *Z*‐scores was observed with increasing steroid doses.

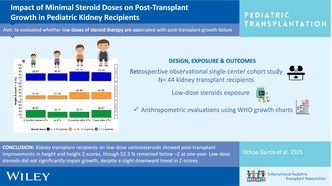

**Abbreviations**
∆: mean differences
CKD: chronic kidney disease
HLA: human leukocyte antigen
IQR: interquartile range
kg: kilogram
KTx: kidney transplantation
NAPRTCS: the North American Pediatric Renal Transplant Cooperative Study
rhGH: recombinant human growth hormone
SD: standard deviation
WHO: World Health Organization

## 1 Introduction

Growth failure is a frequent complication in children with chronic kidney disease stage 5 (CKD stage 5) [1, 2, 3], affecting quality of life and social rehabilitation [1, 2, 4]. Severe growth failure is also associated with a high risk of morbidity and mortality as well as reduced self‐esteem [1, 4, 5, 6, 7].

Although kidney transplantation (KTx) effectively resolves many of the metabolic and endocrine disorders that contributed to growth failure [1, 8], catch‐up growth rarely occurs to achieve normal final adult height [5, 9]. Consequently, approximately 30% to 60% of children with CKD present reduced adult height (height below 1.88 DS or < p3) post‐transplantation [1, 8, 10, 11, 12, 13]. Several factors influence post‐KT growth recovery, including height deficit at the time of KTx, young age at KTx, and graft function [1, 4, 14]. Among modifiable risk factors, data from the North American Pediatric Renal Transplant Cooperative Study (NAPRTCS), which included over 10 000 transplant recipients, identified the use of corticosteroids as a potential contributor to suboptimal growth following transplantation [1, 5]. Previous clinical and observational studies in pediatric KTx recipients have also demonstrated that partial/total glucocorticoid withdrawal is associated with improved growth [4]. Accordingly, corticosteroid‐sparing and corticosteroid withdrawal have been used for post‐transplantation to reduce potential steroid toxicity [4].

Even though the effects of high‐dose steroids on growth are well‐known, steroids are the backbone of most immunosuppressive regimens, and they still play an important role in immunosuppression after solid organ transplantation. Approximately 50% of KTx in pediatric recipients still receive long‐term glucocorticoid therapy [14]. There is a lack of evidence that steroids at minimal doses continue to influence linear growth (low doses commonly referred to as 6 mg/m^2^ or approximately 0.2 mg/kg/day in children) [15]. This study aims to assess if low doses of steroid therapy after KTx are related to persistent growth failure 1 year after KTx.

## 2 Methods

### 2.1 Study Design and Population

This single‐center retrospective longitudinal study was conducted on children who received a kidney transplant from 2005 to 2024 at the Hospital Pablo Tobón Uribe in Medellín, Colombia. The study included children with a first‐time graft, transplanted before the age of 16 years for males and before 14 years for females, with at least two follow‐up visits at 6 months and 1 year after transplantation. Patients with a second kidney transplantation and patients with height‐affecting skeletal abnormalities were excluded.

### 2.2 Data Collection

Demographic, clinical, and outcome information was extracted from electronic health records. This included demographic data such as age, gender, etiology of CKD; history of dialysis, recombinant human growth hormone (rhGH) therapy, and steroid therapy prior to KTx. Clinical data included HLA matching, donor type, graft function, and immunosuppressive therapy used. Anthropometric measurements were collected 6 months before and at 6 and 12 months after KTx. They included total height, weight, and BMI (Body mass index). Height and BMI were converted into *Z*‐scores to adjust for chronological age and sex using the WHO growth charts [16, 17]. Previous studies have demonstrated that chronological age impacts body growth in children with CKD stage 5 [1]. Therefore, measurements were grouped according to age cohorts at KTx (< 11 years and 11 or older). Renal function was evaluated by the Schwartz equation [18].

### 2.3 Outcomes

The primary outcome of this study was the change of height *Z*‐score during the first year post‐KTx. Secondary endpoints included differences in height *Z*‐score before and after kidney transplantation.

### 2.4 Sample Size Calculation

Sample size calculation was carried out based on a previous study by Grohs et al. [1] which evaluated change in body height in 148 prepubertal pediatric kidney transplant recipients. They reported a mean height *Z*‐score of −2.018 (SD 1.08) before and 0.53 2 years after kidney transplantation. Given the one‐year follow‐up in our study, we conservatively assumed a 50% reduction in the observed difference, yielding an expected *Z*‐score change of [(−2.018–0.53)/2] = 1.274. A total of 48 patients, each with three measurements, is required to achieve 80% power at a 5% significance level to detect this difference. The calculation was performed using PASS 2020 software based on a linear mixed model with a random intercept.

### 2.5 Statistical Analysis

Continuous variables were reported as mean and standard deviation (SD) or median and interquartile range according to their distribution. Data distribution was evaluated by investigating kurtosis, skewness, Q‐Q plots, and histograms (Figure S1). Categorical variables were reported as absolute and relative frequency. Repeated measures analysis of variance or Friedman‐Rank tests were used, as appropriate, to compare mean differences (∆) in *Z*‐scores 6 months before, 6 months, and 12 months after kidney transplantation.

Linear mixed models were used to analyze the longitudinal effect steroid therapy on growth outcomes. We included a random intercept to correct for within‐participant correlation. Patients were categorized into three groups based on the distribution of cumulative doses of steroids: lower doses (≤ 33.3rd percentile: 0–0.18 mg/k/day), moderate doses (between the 33.3rd and 66.7th percentiles: > 0.18 to 0.3 mg/k/day), and high doses (> 66.7th percentile: > 0.3 mg/k/day). An interaction term between time and steroid dose was incorporated into the model to evaluate how the rate of change in height varied across different dosage categories during the follow‐up period. Several assumptions were tested prior to carrying out the models. In case of severe deviation from normal distribution and/or homoscedasticity even after log transformation, the nonparametric Analysis for Longitudinal Data “nparLD” was used. We report univariate and multivariate results of the models. Variables found to be significant in univariable analysis (*p* ≤ 0.1) were used in multivariable linear regression models to identify factors associated with ∆ height *Z*‐scores. Survival analysis using the Kaplan–Meier method was conducted to evaluate safety endpoints, specifically acute rejection, graft failure, and mortality.

All reported *p*‐values will be two‐sided and the level of significance in each analysis will be set at 0.05. All statistical analysis will be done using R statistical software version 4.2.

## 3 Results

**FIGURE 1:**
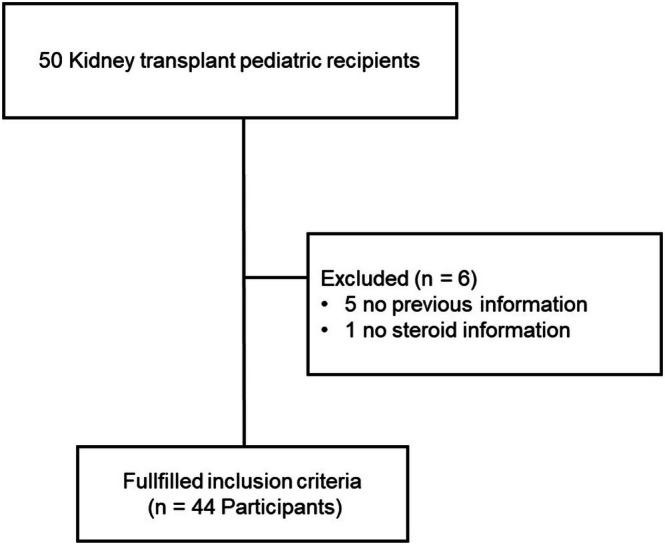
Flow chart total sample included.

Among 50 children who received a KTx between January 2005 and December 2022, six patients were excluded. In total, 44 patients were enrolled and completed 1 year of follow‐up (See Figure 1). The mean age at KTx was 11 years (SD ± 3.8) and 63.6% (*n* = 28) were males. The median time on the wait list was 5 months (p25–75: 2–11.5). Other baseline characteristics are shown in Table 1.

**TABLE 1:** Baseline characteristics evaluated at kidney transplantation.

	Total cohort (*N* = 44)
Gender	
Male *n* (%)	28 (63.6%)
Female *n* (%)	16 (36.4%)
Age (years)	
Mean (±SD)	11.0 (±3.81)
Etiology of CKD	
Unknown *n* (%)	6 (13.6%)
CAKUT *n* (%)	16 (36.4%)
Glomerulopathy *n* (%)	12 (28.3%)
CMV infection *n* (%)	1 (2.3%)
Alport syndrome *n* (%)	1 (2.3%)
Denys Drash syndrome *n* (%)	3 (6.8%)
Prune‐Belly syndrome *n* (%)	2 (4.5%)
Neurogenic bladder *n* (%)	3 (6.8%)
Donor	
Death *n* (%)	43 (97.7%)
Alive *n* (%)	1 (2.3%)
Previous dialysis	
No *n* (%)	13 (29.5%)
Peritoneal *n* (%)	26 (59.1%)
Hemodialysis *n* (%)	5 (11.4%)
Previous steroid therapy	
Yes *n* (%)	6 (13.6%)
rhGH therapy previous	
Yes *n* (%)	5 (11.4%)
Time waits list (months)	
Median (IQR)	5.0 (2.0–11.5)
HLA A matching	
0 *n* (%)	23 (52.3%)
1 *n* (%)	20 (45.5%)
2 *n* (%)	1 (2.3%)
HLA B matching	
0 *n* (%)	30 (68.2%)
1 *n* (%)	13 (29.5%)
2 *n* (%)	1 (2.3%)
HLA DR matching	
0 *n* (%)	24 (54.5%)
1 *n* (%)	20 (45.5%)

Abbreviations: CAKUT, congenital anomalies of the kidney and urinary tract; IQR, interquartile range; rhGH, recombinant human growth hormone.

All patients received pulse doses of methylprednisolone followed by a prednisone taper until low‐dose corticosteroid therapy was achieved (approximately 0.23 mg/kg/day). Additionally, 84% (*n* = 37) received induction therapy. Maintenance immunosuppression agents included a calcineurin inhibitor (cyclosporine or tacrolimus) combined with antiproliferative therapy (mycophenolate or azathioprine). Data on the immunosuppressive regimen are summarized in Table 2. The average prednisolone doses 3 and 6 months after KTx were 0.23 (IQR 0.18–0.41) and 0.21 mg/k/day (IQR 0.14–0.28), respectively (Table 2).

**TABLE 2:** Immunotherapy therapy used.

Variables	Total cohort (*N* = 44)
Induction therapy	
No induction therapy *n* (%)	7 (15.9%)
Thymoglobulin *n* (%)	20 (45.5%)
Alemtuzumab *n* (%)	6 (13.6%)
Basiliximab *n* (%)	10 (22.7%)
Daclizumab *n* (%)	1 (2.3%)
Tacrolimus	
Yes *n* (%)	31 (70.5%)
Ciclosporin	
Yes *n* (%)	11 (25%)
Mycophenolate	
Yes *n* (%)	16 (36.4%)
Azathioprine	
Yes *n* (%)	28 (63.6%)
Steroid dose by month after KTx in mg/k/day median (IQR)	
Steroid doses (first month after KTx)	0.57 (0.42–0.81)
Steroid doses (second month after KTx)	0.31 (0.22–0.57)
Steroid doses (third month after KTx)	0.23 (0.18–0.41)
Steroid doses (fourth month after KTx)	0.22 (0.14–0.43)
Steroid doses (fifth month after KTx)	0.21 (0.14–0.29)
Steroid doses (six months after KTx)	0.21 (0.14–0.28)
Cumulative steroids doses (average including MTP after KTx)^a^ median (IQR)	
Prednisolone equivalence mg/day/SCT^b^	5.93 (4.46–8.39)
Prednisolone in mg/k/day	0.23 (0.15–0.35)

Abbreviation: IQR, interquartile range.

^a^ This steroid dose represents the cumulative doses including methylprednisolone (MTP) bolus after KTx due Acute rejection.

^b^ SCT calculated by Dubois‐Dubois (SCT = 0.20247 × Height^725^ × Weight^0.425^) [19].

### 3.1 Anthropometric Evaluations

At the time of KTx, 65.1% (*n* = 29) of the patients showed impaired linear growth as indicated by a height *Z*‐score ≤ −2. Mean values of height *Z*‐score and BMI *Z*‐score pre KTx were −2.35 (±1.30) and −0.27 (±1.30), respectively. After 1 year, the mean *Z*‐score and BMI *Z*‐score improved to −1.82 (±1.23) and 0.20 (±1.37), respectively (Table 3 and Figure S2). The mean heights at KTx, 6 and 12 months later were 129.28 (±21.42), 131.93 (±20.52), and 134.09 (±19.90), respectively. Additionally, the percentage of patients with short stature (height *Z*‐score ≤ −2) decreased from 65.1% (*n* = 29) at baseline (KTx) to 54.8% (*n* = 24) and 52.3% (*n* = 23) after 6 and 12 months, respectively (Figure S3). In the univariate analysis, a linear mixed model was used to estimate the annual rate of height progression; the mean height growth velocity was 5.37 cm/year (95% CI: 4.50–6.23, *p* < 0.001).

**TABLE 3:** Growth parameter before and after transplantation (*n* = 44).

	6 months before KTx	at KTx	6 months after KTx	1‐year after KTx
Height				
Mean (±SD)	126.31 (±22.05)	129.28 (±21.42)	131.93 (±20.52)	134.09 (±19.90)
Height *Z*‐score				
Mean (±SD)	−2.35 (±1.30)	−2.40 (±1.31)	−2.10 (±1.25)	−1.82 (±1.23)
BMI				
Mean (±SD)	17.11 (±2.56)	16.79 (±2.98)	17.66 (±2.58)	19.00 (±3.92)
BMI *Z*‐score				
Mean (±SD)	−0.27 (±1.30)	−0.69 (±1.37)	−0.23 (±1.16)	0.20 (±1.37)

**FIGURE 2:**
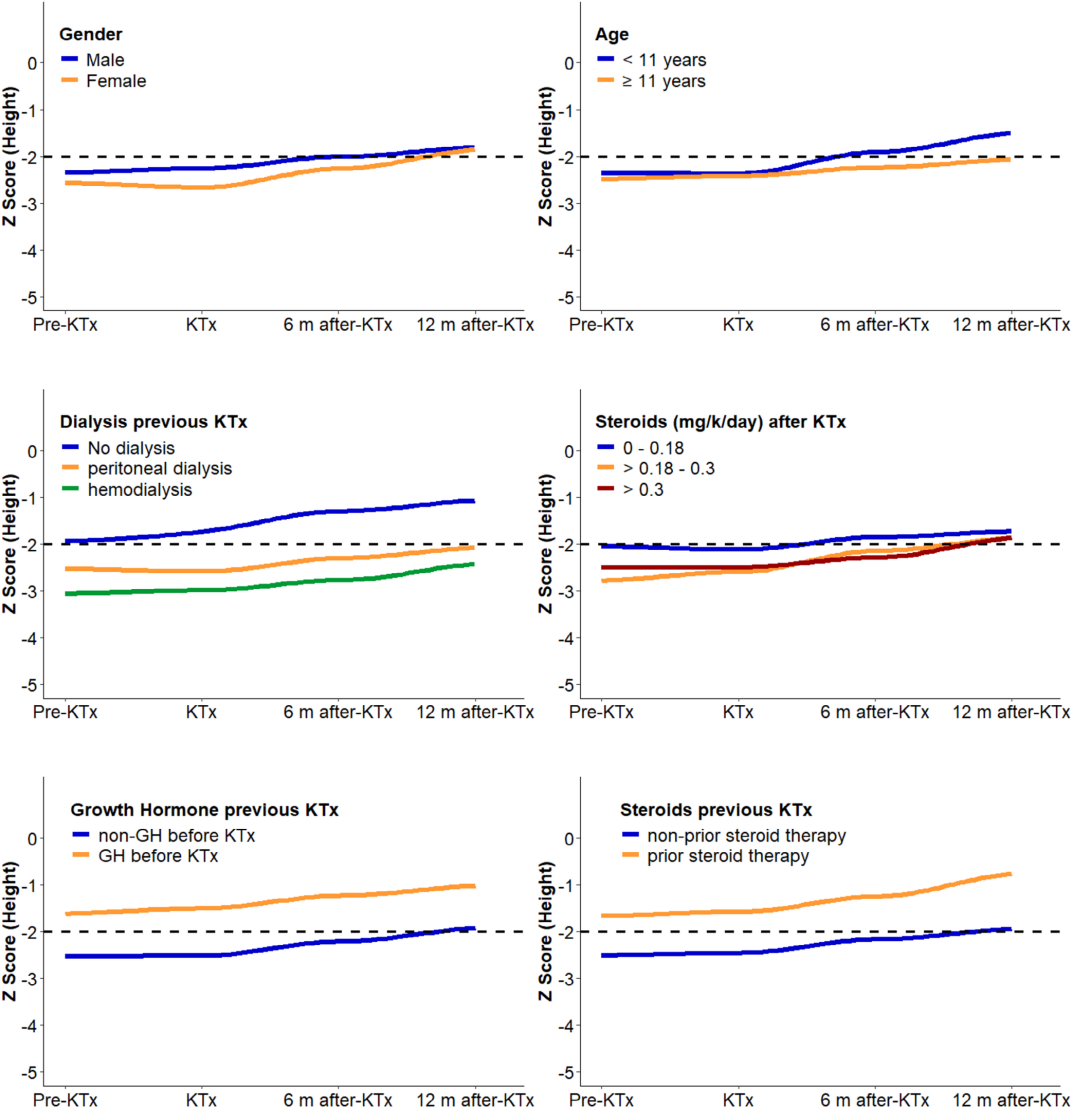
Growth values before and after kidney transplantation. Growth parameters stratified by gender, age, previous dialysis, recombinant human growth hormone therapy before KTx, steroid prior KTx and doses of steroids (mg/k/day) after KTx.

Mean differences (∆) in height *Z*‐score between 6 to 12 months after KTx and 6 months to the time of KTx were greater than the ∆ observed between 6 months before KTx and the time of KTx (*p* < 0.001 and *p* = 0.002 respectively) (Table 4). Figure 2 shows height *Z*‐scores stratified by gender, age, previous dialysis, previous rhGH therapy, and prior comorbidities. This Figure suggests potential variation in growth impairment across subgroups: age at KTx, history of dialysis, and previous therapy with rhGH.

**TABLE 4:** Differences between height and height *Z*‐score.

	Mean ∆_1_	Mean ∆_2_	Mean ∆_3_	*p*
Height difference				
Median [Q1, Q3]	1.80 [0.0, 3.75]	2.25 [1.0, 3.90]	2.90 [2.0, 3.45]	0.725^ft^
Height *Z*‐score difference				
Median [Q1, Q3]	−0.13 [−0.26, 0.28]	0.14 [−0.05, 0.41]	0.22 [0.08, 0.39]	< 0.001^ft^

*Note:* ∆_1_: mean values at KTx—mean values 6 months before KTx; ∆_2_: mean values 6 months after KTx—mean values at KTx, ∆_3_; mean values 6 months after KTx—mean values 12 months after KTx.

Abbreviation: FT, Friedman test.

### 3.2 Relationship Between Cumulative Steroid Doses and Growth

**FIGURE 3:**
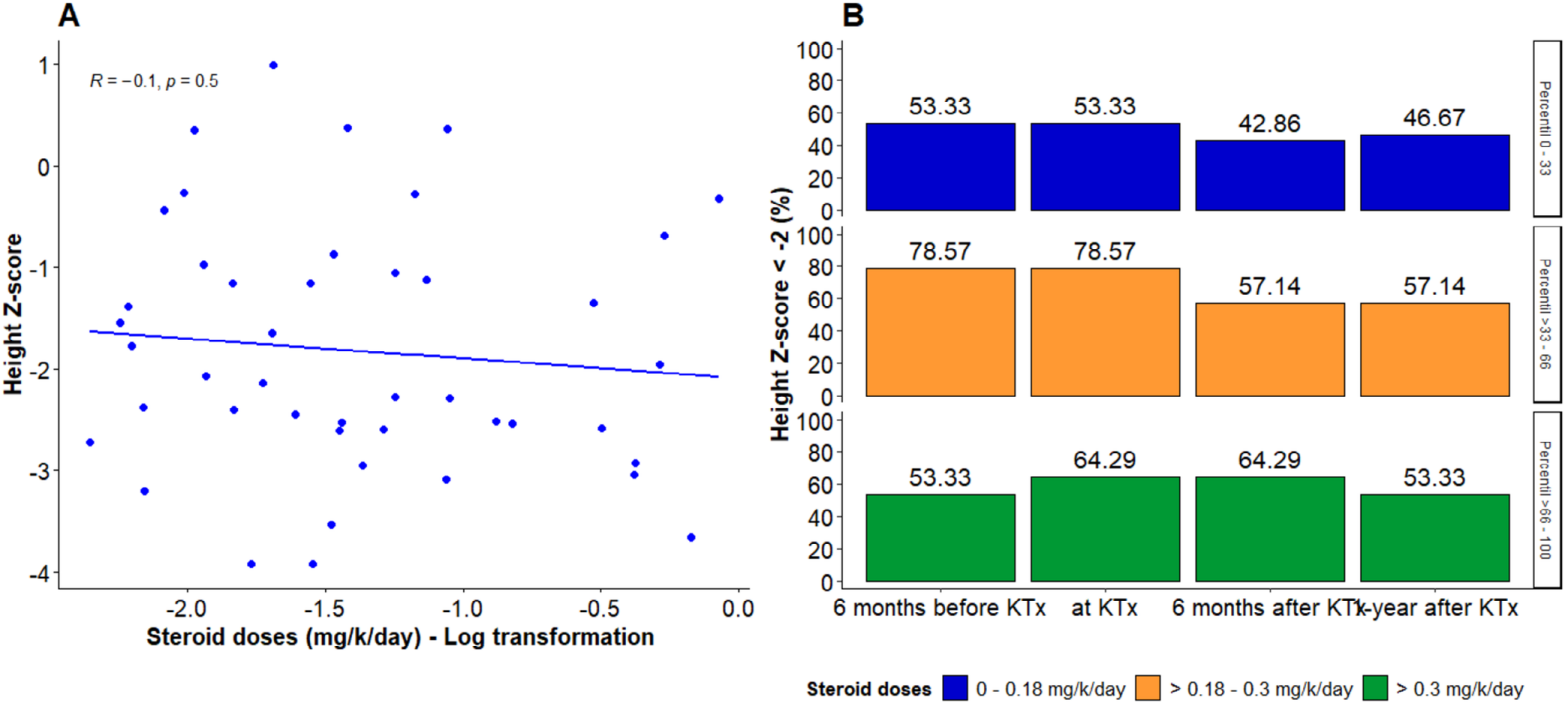
Scatter plot and bar plot showing the relationship between steroid doses and height *Z*‐score. (A) Scatter plot showing height *Z*‐score values on the *y*‐axis and steroid doses (log‐transformed) on the *x*‐axis. (B) Bar plot illustrating the proportion of patients with short stature (height *Z*‐score < −2), stratified by steroid dose categories.

Cumulative doses of steroids used during the first year after KTx were negatively but not significantly correlated with height *Z*‐score (Pearson correlation *r* = −0.10, 95% CI: −0.38 to 0.20) (Figure 3A). Additionally, steroid doses (mg/k/day) were stratified by percentiles 33 and 66, which corresponded to a steroid dose of (1) 0–0.18 mg/k/day; (2) > 0.18–0.3 mg/k/day; and (3) > 0.3 mg/k/day. When stratified by steroid dose, a higher proportion of patients in the group receiving lower doses of steroids achieved a height *Z*‐score greater than −2 (Figure 3B and Figure S3).

### 3.3 Predictors of Linear Body Height

Changes in height *Z*‐score over time were evaluated using a linear mixed model (Table 5). At baseline, there were no differences in height *Z*‐score between the higher steroid dose groups (> 0.18 to 0.3 mg/k/day and > 0.3 mg/k/day) and the lower dose group (0–0.18 mg/k/day). The longitudinal trajectories of height *Z*‐score showed a mean annual decline of 0.53 (95% CI: 0.36–0.71). Patients older than 11 years showed a smaller annual reduction in height *Z*‐score compared with those aged ≤ 11 years (−0.10, 95% CI: −0.15 to −0.06). In contrast, steroid therapy, gender, history of dialysis, and previous therapy with rhGH were not significantly related (Table 5). In the multivariable analysis, steroid therapy (group > 0.18 to 0.3 mg/k/day *p*_interaction_ = 0.12 and group > 0.3 mg/k/day *p*_interaction_ = 0.65) was not significantly associated with either the annual reduction in height *Z*‐score (Table 5) or the height slope (Table S1). Table S2 presents mean values of height and height *Z*‐score stratified by age, sex, and steroid therapy.

**TABLE 5:** Linear mixed‐effect model of *Z*‐score for height.

	Univariate analysis		Multivariate analysis^a^		Interaction *β* (95% CI)	*p* *
	*β* (95% CI)	Interaction *β* (95% CI)	*β* (95% CI)	*p*		
Intercept			−1.41 (−2.42 to −0.39)			
Age in years > 11 years	0.01 (−0.09 to 0.11)	−0.10 (−0.15 to −0.06)	−0.75 (−1.60 to 0.11)	0.10		
Sex female	−0.26 (−1.04 to 0.52)	0.23 (−0.13 to 0.59)				
Previous dialysis	−1.05 (−1.81 to −0.28)	−0.04 (−0.43 to 0.34)				
Previous GH	0.95 (−0.20 to 2.10)	−0.06 (−0.60 to 0.48)				
Steroids after KTX						
0 to 0.18 mg/k/day	ref					
> 0.18 to 0.3 mg/k/day	−0.47 (−1.39 to 0.46)	0.33 (−0.08 to 0.75)	−0.95 (−2.00 to 0.10)	0.09	0.33 (−0.08 to 0.75)	0.12
> 0.3 mg/k/day	−0.27 (−1.17 to 0.64)	0.10 (−0.31 to 0.51)	−0.61 (−1.57 to 0.35)	0.23	0.10 (−0.32 to 0.51)	0.65
Time in years after KTx	0.53 (0.36 to 0.71)		0.39 (0.10 to 0.68)	0.01		

Abbreviation: Ref, reference category.

^a^ IC = 295.85, BIC = 321 Pseudo‐*R* ^2^ (fixed effects) = 0.09.

* *p* value of interaction.

### 3.4 Safety Endpoints: Acute Rejection and Mortality

A total of 6 patients presented with acute rejection episodes. The cumulative incidence of acute rejection at 6‐, and 12‐months post‐kidney transplantation was 9.1% and 13.6%, respectively. One patient experienced graft loss due to arterial obstruction. No mortality was observed during the one‐year follow‐up period.

## 4 Discussion

Linear growth impairment and reduced adult height are well‐documented complications in children with advanced chronic kidney disease. While kidney transplantation (KTx) can enhance growth in pediatric patients, variables such as the age at transplantation, graft function, and the dosage of corticosteroids may adversely affect growth velocity and final adult height [20]. This retrospective single‐center study evaluated the association of low‐dose steroid‐based immunosuppressive therapy on linear growth during the first year following KTx. Our main finding is that post‐transplant growth was superior after KTx, with an increase in the mean standardized height from −2.40 to −1.82 at transplantation and 1 year after KTx.

Corticosteroid therapy is recognized as a negative factor influencing growth following KTx. Evidence indicates that prolonged daily corticosteroid exposure impairs bone growth by affecting the secretion profile of growth hormone [8]. Tourlamain et al. reported that corticosteroid therapy used during the first year post‐KTx was associated with lower height *Z*‐Score values, irrespective of pretransplant rhGH use [21]. Witthenhager et al. assessed the impact of steroid‐free therapy on linear growth in a cohort of 60 pediatric KTx recipients, reporting that 43% of patients had a height *Z*‐score below −2 at KTx, which decreased to 26% one year after KTx [22]. Furthermore, the randomized multicenter TWIST trial demonstrated that early corticosteroid withdrawal improved growth during the first 2 years post‐transplant. In this study, patients treated with tacrolimus, mycophenolate mofetil (MMF), daclizumab with steroid withdrawal at day 4 showed greater increases in height *Z*‐scores compared with those receiving tacrolimus, MMF, and steroid maintenance therapy [23].

Previous studies have also suggested that low, alternate day, or steroid‐free immunosuppressive protocols may be associated with improved post‐transplant growth outcomes [4, 8, 11, 24, 25]. The CRADLE trial, which included 106 pediatric KT recipients, found no statistically significant difference in statural growth 36 months post KT between patients who received standard triple therapy (Tac, MMF, and steroids) and those treated with everolimus, reduced‐dose tacrolimus, and steroid withdrawal at 6 months post‐KTx [26]. In our study, 65.1% of patients presented a height *Z*‐score below −2 at transplantation, decreasing to 52.3% one year post‐KTx. Our findings suggest that minimized steroid exposure may allow growth catch‐up in pediatric kidney transplant recipients.

Previous trials have identified an association between steroid dose and catch‐up growth after KTx [1, 5]. Our study found only a weak non‐significant negative correlation between steroid dose (mg/k/day) and post‐transplant growth (Pearson correlation −0.1). This discrepancy may be attributed to the use of low steroid doses in our cohort, which can reduce the negative impact on growth associated with corticosteroid exposure. Previous studies show similar results. In 2002, Ninik et al. described a cohort of 82 transplant recipients receiving triple immunosuppression therapy (cyclosporine, azathioprine, and steroids) with a decrease in prednisolone doses from 0.64 mg/kg/day in the first month to 0.26 mg/kg/day at 6 months, and a mean of 0.11 mg/kg/day at 7 years. The mean height *Z*‐score improved from −2 at transplantation to −1.5 (range −3.67 to 1.27) at 7 years [8]. Similarly, Englund et al. did not find a correlation between cumulative steroid doses at 5 years and the height *Z*‐score in a cohort of 58 kidney transplant children [10]. In our study, steroid doses showed a trend toward reduced growth following KTx, although this association did not reach statistical significance. These findings suggest that a more vigorous steroid reduction might improve growth outcomes. Nevertheless, it is important to keep in mind that in certain renal transplant scenarios, such as in patients with high immunological risk, complete steroid withdrawal may not be feasible.

Previous studies have identified other factors affecting linear growth after KTx, including age at KTx, bone age, pretransplant height *Z*‐score, graft function, donor type, and time on dialysis [4, 21, 26, 27]. Consistent with these findings, our study also found that younger age at the time of transplantation was associated with increased post‐transplant height.

In our cohort, the cumulative incidence of acute graft rejection at 6‐ and 12‐months post‐KTx was 9.1% and 13.6%, respectively. These rates are comparable to those reported by Woodle et al., who observed a 12‐month biopsy‐confirmed rejection rate of 13% in a cohort of 77 patients receiving basiliximab induction with minimized steroid dosing [28]. Similarly, Rajab et al. reported a 1‐year acute rejection rate of 9.4% using a standard‐dose steroid regimen [29]. In the TWIST study, fewer patients in the steroid‐free group (66/98, 67.3%) than in the TAC/MMF/steroid group (81/98, 82.7%) completed the study per protocol, primarily due to steroid administration for more than 14 days, most often for acute rejection episodes [27].

An important consideration in interpreting growth outcomes post‐transplant is the biological impact of steroids on bone growth. Emerging evidence suggests that corticosteroids may impair bone growth through modulation of the FGF23/FGFR3 signaling pathway, which plays a critical role in chondrocyte proliferation and growth plate function [30]. This effect may contribute to growth impairment even under low‐dose steroid regimens. Although our study did not include mechanistic assessments, integrating such molecular insights could enhance understanding of steroid‐related growth alterations in pediatric KTx recipients. Future prospective studies combining clinical outcomes with mechanistic evaluations are needed to elucidate these complex interactions.

Regarding the use of growth hormone in this cohort, only one patient received it prior to transplantation. The selection of patients who benefit from the use of growth hormone is based on international recommendations and usually does not require prior evaluation by an endocrinologist because in our country nephrologists can prescribe it for the group of patients with CKD. However, not all patients can access this treatment due to health insurance issues.

Our study has some limitations. First, the retrospective design, small sample size, and short follow‐up period limit the assessment of long‐term growth outcomes, including pubertal growth and final adult height. Second, data collected over a 19‐year period may have introduced variability due to changes in treatment protocols and supportive care practices over time. Additionally, important data such as the duration of pre‐transplant rhGH therapy, clinical pubertal status, history of intrauterine growth restrictions, parental height, nutritional intake, and bone age were unavailable. In particular, the absence of bone age assessment limits our ability to explore other variables related to catch‐up growth. Furthermore, our cohort only included one patient with a living donor kidney transplant, which may limit the generalizability of our findings. Therefore, these findings should be regarded as preliminary and hypothesis‐generating, warranting confirmation through larger, prospective, and longer‐term studies.

Strengths: First study in Colombia to evaluate longitudinal growth in post‐renal transplant and its potential relationship with steroid use, which is more in line with current clinical practice where, beyond using or not using steroids, it suggests in some specific contexts using steroids at low doses.

## 5 Conclusion

This single‐center study found that pediatric kidney transplant recipients receiving low‐dose steroid therapy showed improvements in height and height‐for‐age *Z*‐scores post‐transplant. Nevertheless, 52.3% continued to exhibit *Z*‐scores below −2SD in 1 year. Although low‐dose steroid exposure did not appear to substantially impair growth, a slight downward trend in *Z*‐scores was observed over time. Given the study's limited sample size, short follow‐up, and retrospective design, these findings should be interpreted with caution. Larger, prospective studies with longer follow‐up are necessary to confirm the long‐term effects of low‐dose steroid therapy on growth outcomes.

## Author Contributions

All authors contributed to the study conception and design. Material preparation and data collection were performed by O.‐G.C.L. (Ochoa‐García Carolina Lucia), S.‐C.A. (Serna‐Campuzano Angelica), and P.‐O.J.C. (Pelaez‐Ortiz Juan Camilo). Data analysis was performed by S.‐H.L.M. (Serna‐Higuita Lina Maria), I.‐L.M.C. (Isaza‐Lopez Maria Carolina), and V.‐A.E. (Villegas‐Arbelaez Esteban). The first draft of the manuscript was written by O.‐G.C.L., I.‐L.M.C., and S.‐H.L.M. All authors commented on previous versions of the manuscript. All authors read and approved the final manuscript.

## Funding

The authors have nothing to report.

## Ethics Statement

The Ethics Committee approved the study, and the research was performed in accordance with the Declaration of Helsinki.

## Conflicts of Interest

The authors declare no conflicts of interest.

## Supporting information


**Data S1:** Supporting Information.

## Data Availability

The data that support the findings of this study are available on request from the corresponding author. The data are not publicly available due to privacy or ethical restrictions.
