# Supplementary material for: Impact of Minimal Steroid Doses on Post‐Transplant Growth in Pediatric Kidney Recipients, a Retrospective Observational Study
Source: Pediatr Transplant. 2025 Dec 21;30(1):e70251. doi: 10.1111/petr.70251 (PMC12719929; doi:10.1111/petr.70251)
Supplement: Supplementary file 1 — Data S1: Supporting Information. [file PETR-30-e70251-s001.docx]

**Figure 1 Supplemental:** Anthropometric measurements histograms


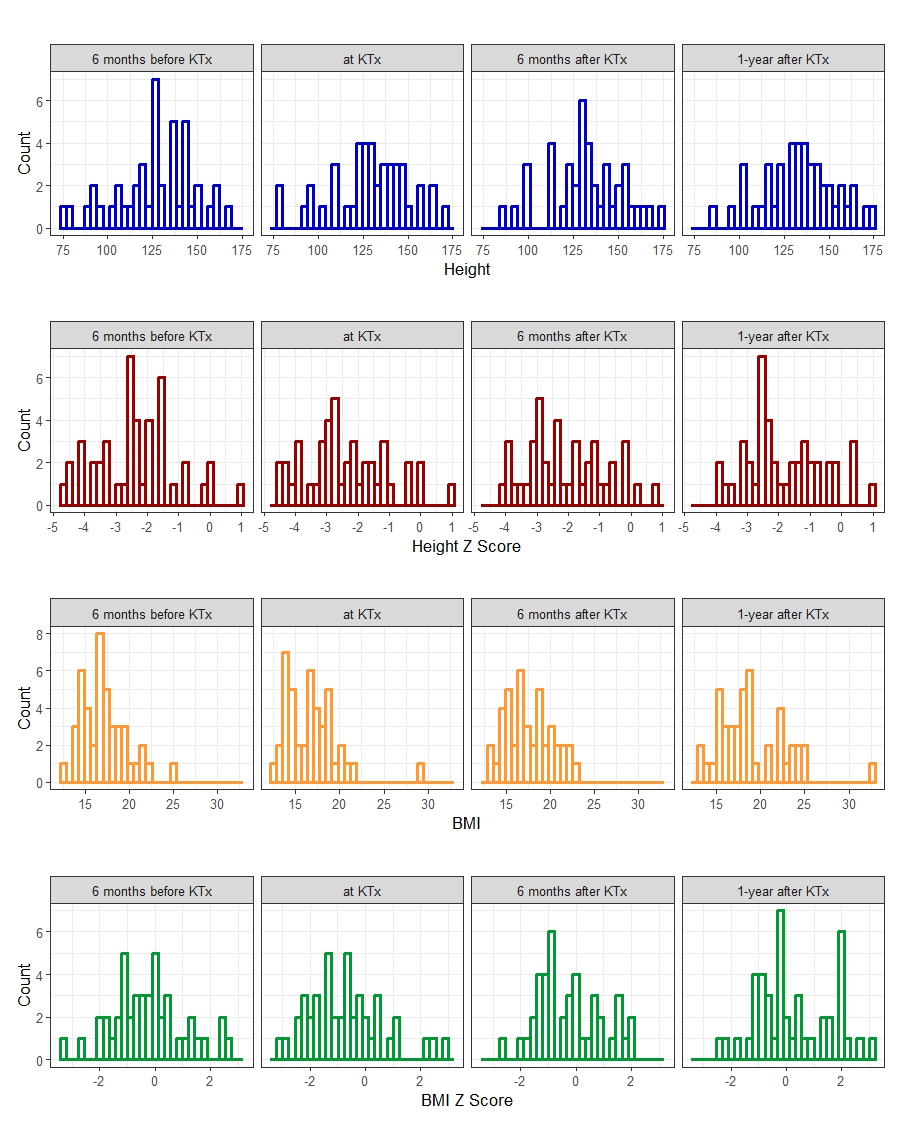


**Figure 2 supplemental:** Median and IQR scores for height, Height Z-score, BMI and BMI Z-score
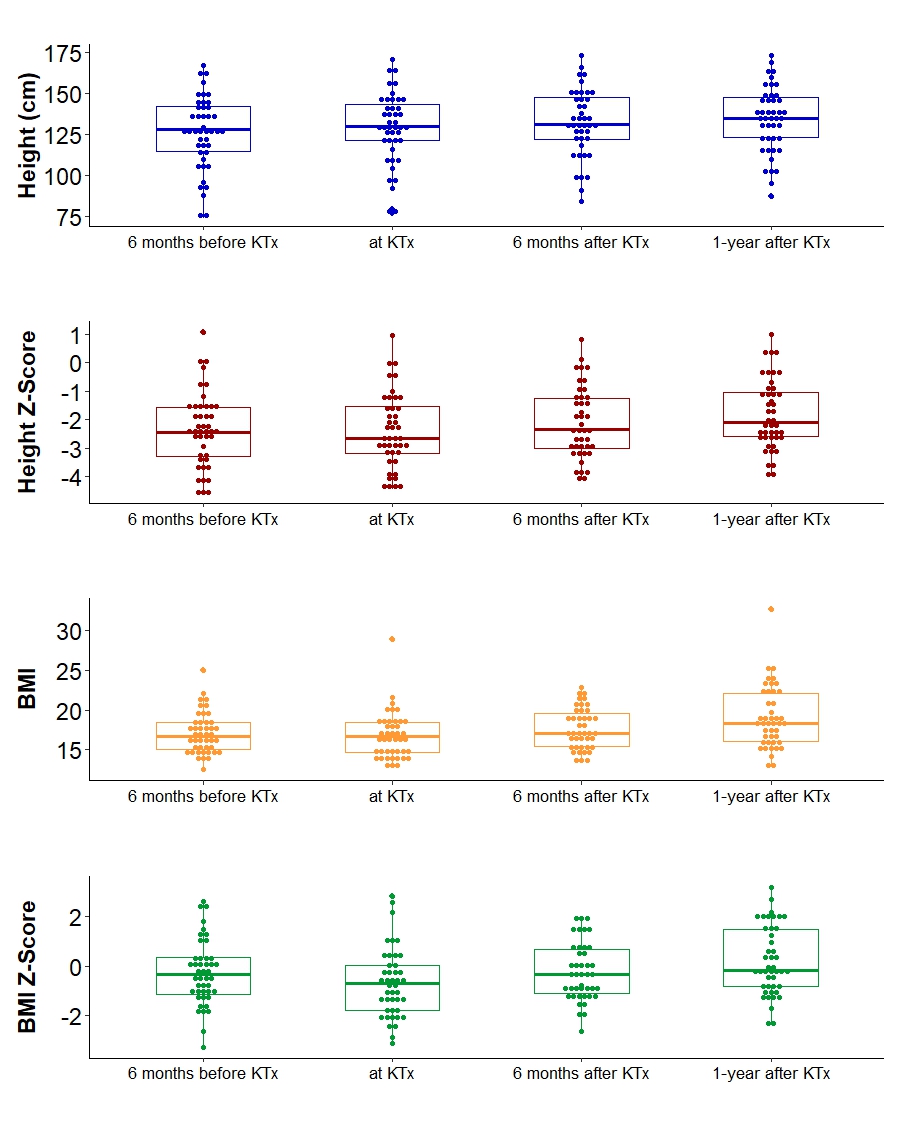


**Figure 3 Supplemental:** Bar plot percentage of patients with short stature after follow-up


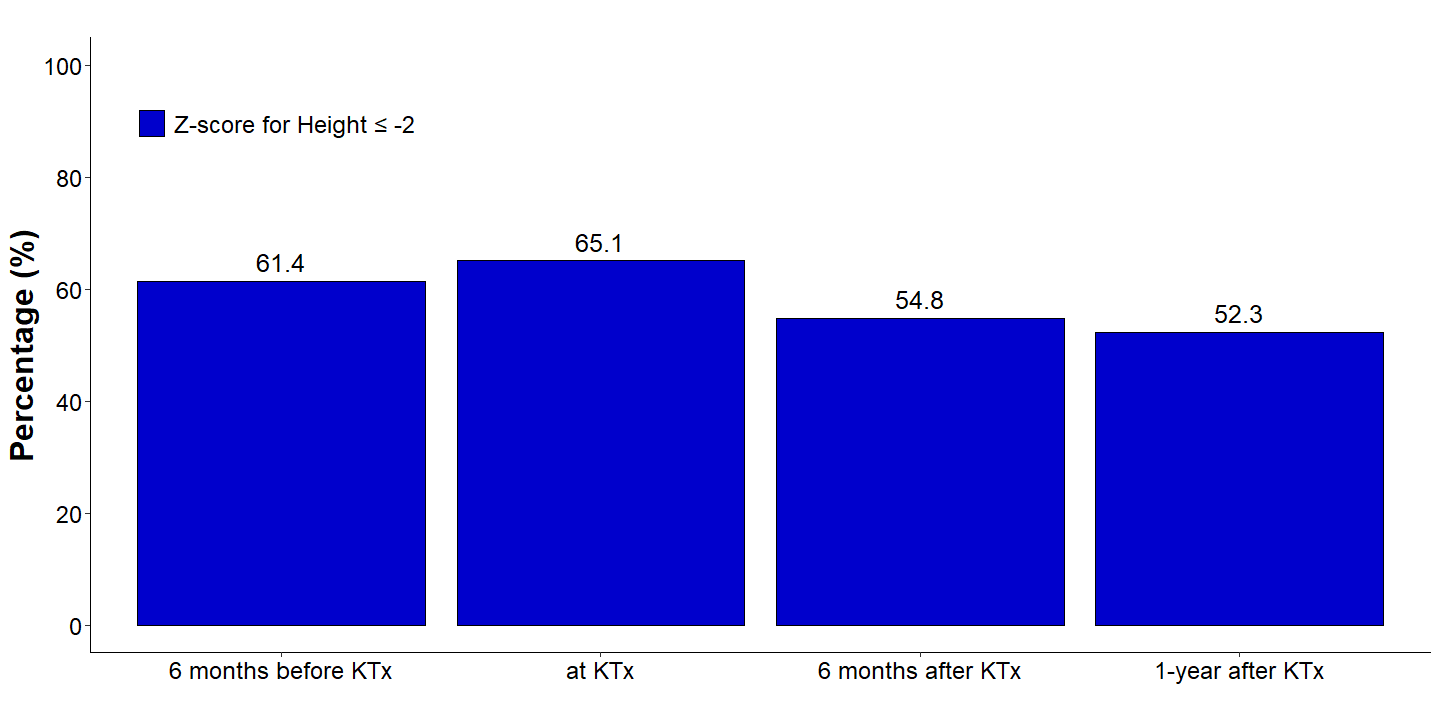


**Table 1 supplemental:** Linear mixed-effect model of body height adjusted by time, steroid therapy and age at KTx

|  | **Univariate Analysis** | | **Multivariate Analysis*** | |
| --- | --- | --- | --- | --- |
|  | **Main Effect**  **β (95% CI)** | **Interaction**  **β (95% CI)** | **Main Effect**  **β (95% CI), p value** | **Interaction**  **β (95% CI), p value** |
| **Intercept** |  |  | 120.7 (109.4 to 131.9) |  |
| Age in years > 11 years | 5.2 (4.5 to 5.8) | -0.42 (-0.6 to -0.2) | 25.7 (16.1 to 35.2), <0.001 |  |
| Sex Female | -1.3 (-14.1 to 11.5) | 0.9 (-0.9 to 2.7) |  |  |
| Previous dialysis | -4.4 (-17.8 to 9.1) | 0.4 (-1.5 to 2.3) |  |  |
| Previous GH | 0.88 (-18.5 to 20) | 0.72 (-1.9 to 3.4) |  |  |
| Steroids after KTX |  |  |  |  |
| 0 to 0.18 mg/k/day | ref |  |  |  |
| > 0.18 to 0.3 mg/k/day | -26.2 (-38.8 to -13.7) | 1.23 (0.85 to 3.31) | -9.63 (-21.2 to 1.96), 0.12 | 1.23 (-0.85 to 3.31), 0.25 |
| > 0.3 mg/k/day | -22.0 (-34.4 to -9.62) | 0.29 (-1.79 to 2.37) | -10.0 (-20.6 to 0.59), 0.08 | 0.30 (-1.79 to 2.38), 0.78 |
| Time in years after KTx | 5.37 (4.5 to 6.23) |  | 4.87 (3.43 to 6.32), <0.001 |  |

*AI*C* = 753, *BIC* = 778 *Pseudo-R² (fixed effects)* = 0.56

**Table 2 supplemental:** Growth parameter previous and after transplantation stratified by gender and age

| **Time** | **Groups** | **Height** | | **Z score Height** | | **BMI** | |
| --- | --- | --- | --- | --- | --- | --- | --- |
|  |  | **mean** | **SD** | **mean** | **SD** | **mean** | **SD** |
| 6-months before | Total cohort | 126.31 | 22.05 | -2.35 | 1.30 | 17.11 | 2.56 |
| Transplantation | Total cohort | 129.28 | 21.42 | -2.40 | 1.31 | 16.79 | 2.98 |
| 6 months after | Total cohort | 131.93 | 20.52 | -2.10 | 1.25 | 17.66 | 2.58 |
| 12 months after | Total cohort | 134.09 | 19.90 | -1.82 | 1.23 | 19.00 | 3.92 |
| *Stratified by age* | |  |  |  |  |  |  |
| 6-months before | <= 11 years | 107.88 | 17.39 | -2.36 | 1.28 | 16.30 | 2.10 |
| 6-months before | > 11 years | 140.32 | 13.08 | -2.34 | 1.35 | 17.72 | 2.75 |
| Transplantation | <= 11 years | 111.41 | 17.78 | -2.37 | 1.33 | 15.63 | 2.39 |
| Transplantation | > 11 years | 142.14 | 12.94 | -2.42 | 1.31 | 17.62 | 3.12 |
| 6 months after | <= 11 years | 114.65 | 15.34 | -1.91 | 1.20 | 16.80 | 2.73 |
| 6 months after | > 11 years | 144.89 | 12.94 | -2.24 | 1.29 | 18.31 | 2.31 |
| 12 months after | <= 11 years | 117.24 | 14.69 | -1.50 | 1.21 | 17.73 | 3.63 |
| 12 months after | > 11 years | 146.89 | 12.34 | -2.07 | 1.21 | 19.97 | 3.92 |
| *Stratified by gender* | |  |  |  |  |  |  |
| 6-months before | Male | 127.01 | 24.19 | -2.23 | 1.32 | 17.55 | 2.84 |
| 6-months before | Female | 125.10 | 18.40 | -2.57 | 1.28 | 16.33 | 1.81 |
| Transplantation | Male | 129.19 | 23.86 | -2.26 | 1.33 | 16.95 | 3.39 |
| Transplantation | Female | 129.43 | 16.70 | -2.66 | 1.26 | 16.47 | 2.05 |
| 6 months after | Male | 131.57 | 23.51 | -2.01 | 1.25 | 17.74 | 2.86 |
| 6 months after | Female | 132.57 | 14.33 | -2.26 | 1.27 | 17.51 | 2.09 |
| 12 months after | Male | 134.25 | 22.63 | -1.81 | 1.18 | 19.42 | 4.31 |
| 12 months after | Female | 133.80 | 14.60 | -1.85 | 1.35 | 18.27 | 3.12 |
| *Stratified by steroid therapy in mg/k/day (percentile 33 and 66)* | | | | |  |  |  |
| 6-months before | [0 - 0.18] | 142.79 | 10.93 | -2.05 | 1.03 | 18.33 | 2.45 |
| 6-months before | [> 0.18 – 0.3] | 116.09 | 20.36 | -2.51 | 1.36 | 16.44 | 2.26 |
| 6-months before | [> 0.3] | 119.38 | 23.28 | -2.50 | 1.51 | 16.51 | 2.65 |
| Transplantation | [0 - 0.18] | 144.47 | 11.23 | -2.12 | 1.08 | 18.59 | 3.22 |
| Transplantation | [> 0.18 – 0.3] | 118.32 | 20.80 | -2.60 | 1.42 | 15.55 | 2.42 |
| Transplantation | [> 0.3] | 123.96 | 22.09 | -2.50 | 1.45 | 16.09 | 2.39 |
| 6 months after | [0 - 0.18] | 147.99 | 11.42 | -1.86 | 1.16 | 19.00 | 2.23 |
| 6 months after | [> 0.18 – 0.3] | 121.51 | 17.84 | -2.15 | 1.38 | 16.58 | 2.48 |
| 6 months after | [> 0.3] | 126.29 | 21.22 | -2.29 | 1.25 | 17.41 | 2.58 |
| 12 months after | [0 - 0.18] | 149.34 | 11.24 | -1.73 | 1.14 | 21.11 | 4.42 |
| 12 months after | [> 0.18 – 0.3] | 124.42 | 17.47 | -1.87 | 1.40 | 17.71 | 3.39 |
| 12 months after | [> 0.3] | 127.86 | 20.56 | -1.87 | 1.22 | 18.11 | 3.12 |
